# Bacteriophage Therapy Against *Shigella* spp.: A Precision Antimicrobial Strategy

**DOI:** 10.3390/antibiotics15030317

**Published:** 2026-03-20

**Authors:** Giuseppe Guido Maria Scarlata, Andrej Belančić, Davor Štimac, Almir Fajkić, Tomislav Meštrović, Ludovico Abenavoli

**Affiliations:** 1 Department of Health Sciences, University “Magna Graecia”, 88100 Catanzaro, Italy; l.abenavoli@unicz.it; 2 Center for Chronic Liver Diseases, “Renato Dulbecco” University Hospital, 88100 Catanzaro, Italy; 3 Department of Basic and Clinical Pharmacology with Toxicology, Faculty of Medicine, University of Rijeka, Braće Branchetta 20, 51000 Rijeka, Croatia; andrej.belancic@uniri.hr; 4 Department of Gastroenterology, Clinical Hospital Centre Rijeka, 51000 Rijeka, Croatia; davor.stimac@uniri.hr; 5 Department of Pathophysiology, Faculty of Medicine, University of Sarajevo, 71000 Sarajevo, Bosnia and Herzegovina; 6 University Centre Varaždin, University North, 42000 Varaždin, Croatia; tmestrovic@unin.hr; 7 Institute for Health Metrics and Evaluation, University of Washington, Seattle, WA 98105, USA

**Keywords:** infection, treatment, management, precision medicine

## Abstract

Shigellosis remains a significant global cause of infectious colitis, increasingly complicated by multidrug-resistant strains and the microbiota-disrupting effects of broad-spectrum antibiotics. Although conventional antimicrobial therapy can reduce symptom duration and bacterial shedding, it also contributes to gut dysbiosis, loss of colonization resistance, and further selection for antimicrobial resistance. These challenges have renewed interest in precision antimicrobial strategies, particularly bacteriophage therapy, which provides strain-level specificity and preserves the gut microbiota. This narrative review evaluates the biological rationale, preclinical and early clinical evidence, safety considerations, and translational challenges associated with bacteriophage therapy targeting *Shigella* spp. The historical development and mechanistic basis of phage therapy are summarized, with emphasis on the advantages of obligately lytic phages, receptor-specific targeting, self-amplification at infection sites, and activity against both planktonic and biofilm-associated bacteria. Recent microbiota research indicates that shigellosis is closely associated with early and persistent disruption of gut ecology, including depletion of short-chain fatty acids-producing taxa and reduced microbial resilience. Phage-based approaches may reduce pathogen burden while preserving beneficial microbial communities. Evidence from in vitro systems, animal models, human intestinal organoids, and a Phase 1 clinical trial demonstrates targeted efficacy and favorable safety profiles for *Shigella*-specific phages and phage cocktails. Major barriers to clinical adoption include immune interactions, phage resistance dynamics, genomic safety screening, regulatory classification, and the need for standardized susceptibility testing. Future directions emphasize the development of personalized phage therapy platforms that integrate rapid diagnostics, phage libraries, metagenomics, and artificial intelligence-assisted matching to enable scalable, precision treatment.

## 1. Introduction

Shigellosis is a major cause of infectious colitis worldwide and remains a significant public health concern, particularly in low- and middle-income countries, but with increasing relevance in high-income settings due to global travel, urban crowding, and antimicrobial resistance (AMR) [1,2,3]. Caused by bacteria of the genus *Shigella*, the disease is characterized by acute inflammation of the colonic mucosa, leading to symptoms such as bloody diarrhea, abdominal pain, fever, and tenesmus [4]. Even a low infectious dose is sufficient to cause disease, making *Shigella* highly transmissible and responsible for frequent outbreaks in both community and institutional settings [2]. In vulnerable populations, including children, the elderly, and immunocompromised individuals, shigellosis can result in severe complications and increased mortality [1].

Historically, antibiotic therapy has played a central role in the management of shigellosis by shortening disease duration, reducing bacterial shedding, and limiting transmission. However, the rapid emergence and global spread of multidrug-resistant (MDR) *Shigella* spp. have substantially undermined the effectiveness of conventional antimicrobial regimens. AMR has been documented against first-line agents such as ampicillin, trimethoprim-sulfamethoxazole, and fluoroquinolones, as well as against third-generation cephalosporins and macrolides in some regions. This escalating resistance crisis has narrowed therapeutic options and complicated empirical treatment strategies, particularly in severe or refractory cases [3,5,6].

Beyond AMR, antibiotic therapy for shigellosis is associated with inherent clinical and microbiological limitations. Broad-spectrum antibiotics disrupt the gut microbiota, potentially exacerbating gut dysbiosis, prolonging recovery, and increasing susceptibility to secondary infections. In addition, antibiotic exposure exerts selective pressure that further drives resistance development, not only in *Shigella* but also among commensal gut bacteria [7]. These challenges highlight the need for alternative approaches that are both effective against the pathogen and minimally disruptive to the host microbial ecosystem.

Precision antimicrobial strategies aim to address these shortcomings by selectively targeting pathogens while preserving beneficial commensal bacteria. In this context, bacteriophage therapy has re-emerged as a promising and highly specific antimicrobial treatment. Bacteriophages are viruses that infect bacteria; lytic phages, in particular, induce bacterial lysis, whereas temperate phages may establish lysogeny. Their narrow host specificity enables targeted eradication of *Shigella* strains while preserving the surrounding microbial community. However, despite encouraging evidence, significant knowledge gaps remain regarding optimal phage selection, dosing strategies, and long-term safety, which currently limit widespread clinical adoption. Their capacity to self-amplify at the site of infection and to adapt to bacterial resistance mechanisms further enhances their therapeutic potential and positions them as innovative strategies to combat MDR enteric pathogens such as *Shigella* [8,9,10].

This narrative review evaluates the biological rationale, preclinical and early clinical evidence, safety considerations, and translational challenges associated with bacteriophage therapy targeting *Shigella* spp.

## 2. Materials and Methods

A targeted literature search was conducted using PubMed, Scopus, and Web of Science covering the time span from January 2015 to December 2025, with exceptions for historical references relevant to the development of phage therapy. Key search terms included “*Shigella*”, “bacteriophage therapy”, “lytic phage”, “biofilm”, and “microbiota”. Studies were further explored and discussed if they evaluated strictly lytic phages against *Shigella* spp. in vitro, in animal models, human organoids, or clinical trials, prioritizing clinical evidence while also incorporating preclinical and translational studies to provide a mechanistic and therapeutic context.

## 3. Historical Perspective and Biological Basis of Bacteriophage Therapy

**Figure 1:**
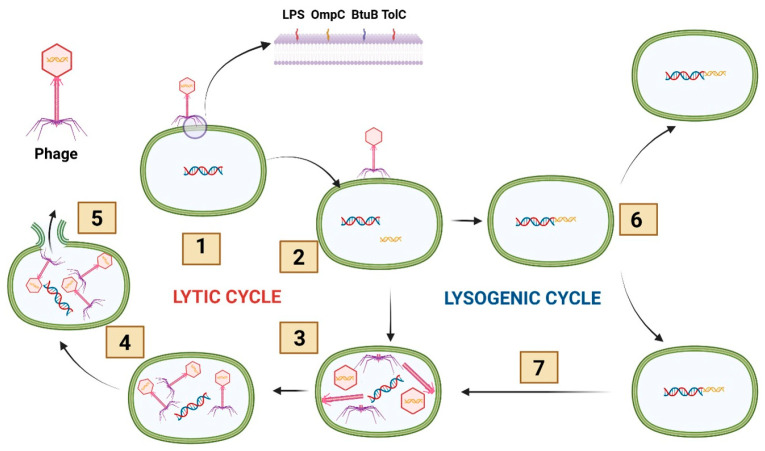
Bacteriophage infection of bacteria—lytic and lysogenic pathways. (1) Attachment (adsorption): the bacteriophage binds to bacterial surface receptors, including LPS and outer membrane proteins (OmpC, BtuB, and TolC). (2) Penetration: the phage injects its genome into the host cell. (3) Biosynthesis: phage DNA hijacks the host machinery to replicate its genome and synthesize phage proteins. (4) Maturation (assembly): newly synthesized phage components are assembled into complete virions within the host cell. (5) Lysis and release: the bacterial cell is lysed, and progeny phages are released. (6) Integration (lysogeny): alternatively, phage DNA integrates into the bacterial chromosome as a prophage. (7) Prophage replication: the prophage is replicated with the host genome during bacterial cell division and can later be induced to re-enter the lytic cycle. Abbreviations: LPS, lipopolysaccharide.

Phage therapy is not a “new” antimicrobial concept but an older strategy returning with modern justification. Targeted phage therapy emerged from early 20th-century microbiology after the recognition that bacteriophages are viruses capable of infecting and lysing bacterial cells. Following Twort’s observation of transmissible bacterial lysis (1915) and d’Hérelle’s characterization of phages (1917), therapeutic use was pursued early, with clinical applications reported soon after discovery [11,12]. While interest declined in Western medicine after Fleming’s discovery of penicillin (1928) and the rapid expansion of antibiotics in the 1940s, phage practice persisted in Eastern Europe and the Soviet sphere, supported by institutional pipelines for isolation, selection, and production, exemplified by the Eliava Institute in Tbilisi [13,14]. Antibiotics ultimately dominated because they were easier to mass-produce, standardize, and prescribe empirically across syndromes [13,14]. This historical divergence is being revisited under a more urgent framing. Antibiotic pressure is reshaping enteric ecosystems, selecting for MDR pathogens while weakening colonization resistance. In gastroenterology and hepatology, this translates into dysbiosis-linked recurrence, bacterial translocation, and difficult complications in fragile hosts [15]. The accelerating burden of MDR organisms—including methicillin-resistant *Staphylococcus aureus*, *Pseudomonas aeruginosa*, *Klebsiella pneumoniae*, and *Acinetobacter baumannii*—has reinvigorated interest in phage therapy as a targeted antimicrobial strategy [16]. High-profile compassionate-use experiences, including the personalized phage cocktail administered in a life-threatening *Acinetobacter baumannii* infection, have further strengthened the clinical plausibility of individualized phage interventions [11,16]. Biologically, therapy begins with lifecycle choice (Figure 1). Phages interact with bacterial hosts mainly through lytic and lysogenic programs. In the lytic cycle, phages bind to bacterial surfaces via receptor-binding proteins on tail fibers, enabling recognition of specific receptors such as lipopolysaccharide (LPS) components or outer membrane proteins (for example, OmpC in *Escherichia coli*, BtuB, and TolC) [17,18]. After adsorption, the phage injects its genome, hijacks host machinery to generate progeny virions, and lyses the cell to release new phages [19]. Temperate phages can instead enter lysogeny, integrate as prophages, and persist without immediate killing, raising concerns about horizontal gene transfer in dense, genetically plastic gut communities. For this reason, clinical development generally prioritizes obligately lytic phages and strict genomic screening [20]. Killing mechanisms are being increasingly mapped in detail, particularly for Gram-negative enteropathogens. Lysis commonly proceeds as staged envelope disruption: holins create inner-membrane lesions that permit endolysins to degrade peptidoglycan, while spanins complete outer membrane disruption to enable virion release. Intestinal conditions can shift these kinetics: growth state, bile exposure, osmotic gradients, and biofilm architecture influence access and replication [21]. Some phages also deploy “single-gene lysis” strategies that block cell wall synthesis rather than enzymatic peptidoglycan degradation; the Phi X174 protein E inhibition of MraY is a canonical example [22]. Efficacy in vivo is also ecological. In the gut, mucus, crypt microhabitats, and biofilm refuges can shield bacteria and impose amplification thresholds, so strong in vitro lysis may not translate if the phage cannot access the relevant niche [23]. These features underpin key advantages over conventional antibiotics. Strain-level specificity can limit off-target gut microbiota depletion and preserve colonization resistance, a priority in patients at risk of recurrent infection or antibiotic-associated complications [24]. Activity can increase where the target expands because replication couples to susceptible bacterial density, while non-susceptible communities are largely spared [25]. AMR is expected but manageable through rational cocktails, iterative matching, and combination regimens; escape via receptor modification may impose fitness costs [25]. Together, these properties position phage therapy as an adaptive antimicrobial platform with particular appeal in gastroenterology and hepatology, where microbial ecology and antimicrobial pressure strongly determine outcomes. However, important biological limitations must also be considered. The emergence of phage-resistant bacterial populations is a predictable consequence of phage-host coevolution and may occur through receptor modification, phase variation, or CRISPR-Cas-mediated defense mechanisms that impair phage adsorption or replication [26]. Although such adaptations may impose fitness trade-offs, they can reduce therapeutic efficacy if not addressed through rational cocktail design or adaptive phage selection strategies. In addition, host immune responses play a critical role in shaping phage pharmacokinetics and therapeutic activity. Circulating phages may be cleared by the mononuclear phagocyte system and neutralized by anti-phage antibodies, potentially limiting persistence and antibacterial effectiveness [27]. At the intestinal level, mucus layers and mucosal immune components influence phage localization and access to bacterial targets, while also contributing to immune recognition and modulation of phage-host interactions [28]. These biological constraints underscore the importance of integrating evolutionary and immunological considerations into the design of effective phage therapy strategies, particularly in complex gastrointestinal environments.

## 4. Gut Microbiota and *Shigella* Infection

Acute infectious diarrhea is increasingly recognized as a state of rapid gut microbiota disruption that can arise early in illness, often before any antimicrobial exposure [29,30,31]. Studies in pediatric populations from high-burden settings show marked alterations in microbial community structure, characterized by depletion of obligate anaerobes and enrichment of facultative anaerobic taxa within days of symptom onset [31]. These changes are heterogeneous, with microbiota configurations ranging from near-healthy states to pronounced dysbiosis dominated by bacterial genera such as *Escherichia* and *Streptococcus*, and are shaped by host factors including age, nutritional status, breastfeeding practices, and infectious etiology [29,31].

Additional evidence from adult travel-associated diarrhea further supports a consistent pattern of gut microbiota disruption during enteric exposure, marked by depletion of microbial diversity and expansion of Enterobacteriaceae that frequently persist beyond the acute episode and interact with AMR dynamics [32].

**Figure 2:**
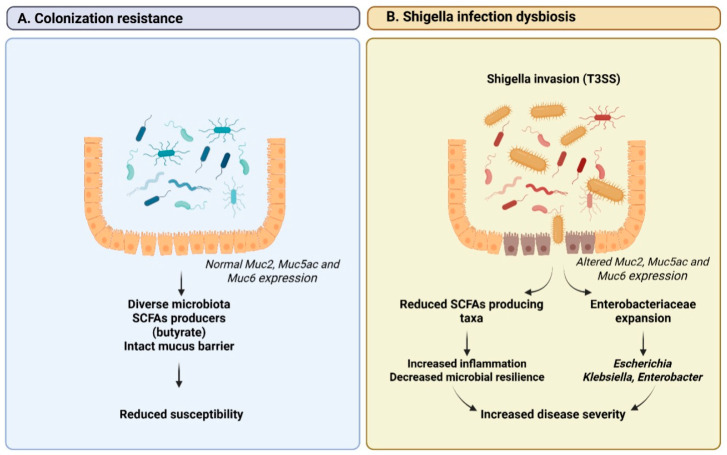
Colonization resistance and gut microbiota dysbiosis during *Shigella* infection. (**A**) Colonization resistance is maintained by a diverse gut microbiota enriched in SCFAs-producing taxa (particularly butyrate producers) together with an intact mucus barrier. Normal MUC2, MUC5AC, and MUC6 expression supports mucus layer integrity, limits bacterial–epithelial contact, and reduces susceptibility to infection. (**B**) *Shigella* infection is associated with T3SS-mediated invasion, altered MUC2, MUC5AC, and MUC6 expression, and mucus barrier disruption. These changes coincide with depletion of SCFAs-producing taxa and expansion of Enterobacteriaceae (e.g., *Escherichia*, *Klebsiella*, and *Enterobacter*), leading to increased inflammation, reduced microbial resilience, and greater disease severity. Abbreviations: SCFAs, short-chain fatty acids.

In that context, shigellosis is characterized by invasive intestinal inflammation, but also by profound disturbances of the gut microbial ecosystem [33]. The interplay between *Shigella* spp. and resident microbiota plays a pivotal role in disease susceptibility, clinical severity, and, subsequently, recovery [33]. Recent genetic epidemiological evidence supports a specific pattern of bidirectional relationship, demonstrating that specific gut microbial taxa exert potentially causal effects on the risk of *Shigella* infection; moreover, *Shigella* itself can induce downstream alterations in microbiota composition [34]. In particular, reduced abundance of butyrate-producing and anti-inflammatory genera has been associated with increased susceptibility, while enrichment of taxa linked to epithelial permeability and inflammatory signaling correlates positively with infection risk [34]. It was shown that *Shigella* infection can drive measurable shifts in microbial community structure, reinforcing gut dysbiosis beyond the acute phase and the reciprocal nature of host-microbiota-pathogen interactions in shigellosis, as reported in Figure 2 [34].

Experimental models indeed demonstrate that *Shigella* infection induces route- and severity-dependent disruption of the gut microbiota. In mice, oral challenge with *Shigella flexneri* has been shown to cause rapid but largely transient compositional shifts, marked by early depletion of *Lactobacillus* and expansion of Prevotellaceae and *Escherichia/Shigella*, with minimal impact on overall α-diversity [35]. More severe infection was associated with delayed, yet pronounced loss of microbial diversity and sustained ecological disturbance [35]. Across infection routes, reduction of short-chain fatty acids (SCFAs)-producing and immunomodulatory taxa accompanied disease progression, underscoring the close linkage between inflammatory burden and gut microbiota stability during shigellosis [34,35].

Evidence from a controlled human infection model demonstrates that individuals who develop clinical shigellosis experience a significantly greater reduction in microbial α-diversity during infection, accompanied by marked divergence in overall community composition when compared with exposed individuals who remain asymptomatic [36]. These changes are not solely attributable to increased *Shigella* abundance but reflect broader ecological shifts, including depletion of taxa linked to gut health and SCFAs production (such as *Faecalibacterium*, *Roseburia,* and *Bifidobacterium*), alongside enrichment of inflammation-associated organisms (such as *Ruminococcus gnavus*) [36]. Notably, gut microbiota disruption in shigellosis emerges early in the course of infection and, in contrast to asymptomatic exposure, frequently persists beyond clinical recovery, indicating impaired microbial resilience [36]. Baseline gut microbiota composition also appears to modulate susceptibility, with higher pre-infection abundance of butyrate-producing taxa associated with reduced risk of symptomatic disease [36].

Longitudinal and cross-sectional studies from diverse low- and middle-income settings are consistent in demonstrating how *Shigella* infection can be embedded within broader, age- and context-dependent microbiota dynamics, rather than representing an isolated pathogen-driven event [37,38,39]. A longitudinal birth cohort study from Malawi showed that, while overall gut microbial diversity increased with age regardless of infection status, *Shigella* infection was associated with distinct temporal shifts in community composition [37]. These include post-infection enrichment of taxa from the family *Lachnospiraceae*, such as *Fusicatenibacter saccharivorans* and *Lachnospiraceae* NK4A136, which are SCFAs producers potentially involved in microbial recovery and restoration of gut homeostasis [37]. Complementing these findings, analysis of stool samples from the Global Enteric Multicenter Study revealed that susceptibility to shigellosis is influenced more by baseline microbiota structure rather than pathogen burden alone, with higher bacterial diversity and the presence of specific *Lactobacillus* taxa associated with reduced diarrheal risk, even in the presence of high *Shigella* loads, highlighting the role of colonization resistance and protective microbial interactions [38]. More recently, metagenomic profiling of diarrheal children from multiple low- and middle-income countries demonstrated that *Shigella*-positive cases exhibit a distinct dysbiotic signature characterized by enrichment of Proteobacteria and depletion of *Bifidobacterium*, as well as co-enrichment of virulence and AMR gene modules linked predominantly to Enterobacteriaceae [39]. This points toward functional reorganization of the gut ecosystem that extends beyond taxonomic shifts and may influence both pathogenicity and diagnostic detectability.

Considering such an impact on the gut microbiota, as well as the central role of microbial resilience and SCFAs-producing taxa in modulating susceptibility and recovery, interventions that minimize collateral disruption of the gut ecosystem may offer advantages over broadly acting antimicrobial agents [40]. Such approaches can be especially pertinent for high-burden settings and younger age groups, where repeated antibiotic exposure and delayed microbiota recovery may amplify gut dysbiosis and long-term vulnerability to enteric infection. Bacteriophage therapy in this context represents a targeted, microbiota-sparing antimicrobial strategy capable of selectively eliminating *Shigella* while largely preserving commensal gut bacteria [41]. By reducing pathogen burden without the broad ecological disruption associated with antibiotics, phage-based approaches may support colonization resistance and limit downstream dysbiosis during and/or after infection [40,42].

## 5. Bacteriophage Therapy Targeting *Shigella* spp.

As previously reported, the genus *Shigella* comprises four closely related species (*S. dysenteriae*, *S. flexneri*, *S. boydii*, and *S. sonnei*) that cause shigellosis, an acute inflammatory colitis characterized by epithelial invasion, mucosal ulceration, and intense innate immune activation [43]. A distinctive feature of *Shigella* pathogenesis is its extremely low infectious dose, which reflects a high degree of adaptation to the gastrointestinal environment, resistance to gastric acidity, and efficient mechanisms of epithelial adherence, invasion, and intracellular spread [44]. These features allow *Shigella* to rapidly establish infection despite host defense mechanisms and competing commensal microbiota. While these characteristics confer marked virulence, they also define specific biological dependencies that render *Shigella* spp. particularly amenable to bacteriophage-based interventions [45].

At the structural level, *Shigella* expresses several surface-exposed components that are directly involved in pathogenicity. These include LPS O-antigens, outer membrane proteins, and polysaccharide structures. These elements mediate epithelial attachment and invasion. They are critical for host–pathogen interactions and are often exploited by lytic bacteriophages as receptors for adsorption and entry [46]. Many of these receptors are essential for bacterial fitness and intestinal colonization. As a result, bacterial escape from phage predation through receptor modification or loss may reduce virulence or competitive ability within the gut. This evolutionary trade-off is highly relevant to AMR. It contrasts with AMR mechanisms, which may preserve or even enhance pathogenic potential [47].

*Shigella* demonstrates a notable capacity for biofilm formation, especially under stress. Biofilm-associated growth aids bacterial persistence, antibiotic tolerance, and prolonged fecal shedding. It has been linked to treatment failure and recurrent infection [48]. Biofilms pose a significant limitation to conventional antimicrobial therapy, as antibiotics are less effective within bacterial communities. Several *Shigella*-specific bacteriophages, however, possess enzymes such as depolymerases. These allow penetration and disruption of biofilm matrices, enhancing bacterial clearance and reducing persistence [49]. The ability to target both free-living and biofilm-associated bacteria supports the plausibility of phage therapy in shigellosis.

A growing body of preclinical evidence supports the efficacy of bacteriophages against *Shigella* spp. in vitro and in animal models. Notably, early murine studies established that orally administered phage cocktails can significantly reduce intestinal colonization and fecal shedding of *Shigella* with no measurable toxicity or long-term effects on the gut microbiota. In one key study performed by Mai et al., phage treatment led to a rapid and sustained reduction in *Shigella* burden, comparable to ampicillin, while at the same time preserving gut microbial diversity, unlike antibiotic treatment [50]. In this murine model, the bacteriophage cocktail consisted of five obligately lytic bacteriophages (SHSML-52-1, SHFML-11, SHSML-45, SHFML-26, and SHBML-50-1) administered orally at a dose of approximately 1 × 10^9^ plaque-forming units (PFU) per mouse in 0.1 mL via gavage. Treatment was evaluated using multiple dosing regimens, including administration 1 h before bacterial challenge, 1 h after challenge, 3 h after challenge, or both 1 h before and 1 h after challenge. The double-dose regimen demonstrated the greatest efficacy, reducing fecal bacterial counts from 1114 CFU/pellet in untreated mice to 26 CFU/pellet at 24 h post-infection. Additionally, repeated administration of 1 × 10^9^ PFU twice a day for 7 days showed sustained safety and microbiological stability, with no detectable toxicity or microbiota disruption. These findings demonstrated that both dose magnitude and timing relative to bacterial exposure critically influence therapeutic efficacy, and that sufficient phage titers are required to achieve effective bacterial suppression and maintain phage activity in the intestinal environment.

Building on these foundational studies, subsequent investigations isolated and characterized novel lytic phages active against multiple *Shigella* spp. and serotypes. For example, Mondal et al. described Sspk23, which demonstrated strong in vitro activity against *S. sonnei*. Detailed phenotypic analyses showed efficient bacterial lysis across a range of multiplicities of infection and the ability to disrupt established *Shigella* biofilms. Cytotoxicity assays on human intestinal epithelial cells and macrophage-like cell lines revealed no evidence of host cell toxicity, supporting the biological safety of the phage preparation [51]. These data reinforce bacteriophages’ selective targeting of pathogenic *Shigella* populations and extend preclinical findings to broader serotypes.

More recently, a comprehensive study by the same research group described the isolation of the lytic phage SSG23, which has a broad host range encompassing all four major *Shigella* spp. (*S. dysenteriae*, *S. flexneri*, *S. boydii*, and *S. sonnei*). This phage displayed remarkable stability across a wide range of pH and temperature conditions, an essential feature for oral administration in the gastrointestinal tract. Whole-genome sequencing confirmed the absence of genes associated with toxin production, lysogeny, or AMR, addressing key safety concerns related to phage therapy. In *S. sonnei*-infected BALB/c mice, oral administration of SSG23 resulted in a significant reduction in intestinal colonization and fecal shedding, accompanied by improvement in clinical parameters. Although neutralizing antibodies developed following repeated exposure, phage efficacy was preserved throughout the treatment period, suggesting that immune recognition did not abrogate therapeutic activity within the gut lumen [52].

Beyond preclinical animal models, advanced human-relevant experimental systems have been increasingly employed to further assess phage-*Shigella* interactions and to enhance translational relevance. Human intestinal organoid-derived epithelial monolayers recapitulate key features of the intestinal barrier, including cellular polarization, mucus secretion, and innate immune signaling pathways. Using this system, Llanos-Chea et al. demonstrated that a *Shigella flexneri*-specific bacteriophage efficiently killed the pathogen and, critically, prevented epithelial adherence and invasion. Notably, the phage retained strict specificity for *Shigella* strains and did not affect commensal *Escherichia coli* in co-culture, reinforcing the concept of precision targeting [53]. These mechanistic insights highlight how phage therapy can potentially interrupt early steps of *Shigella* infection in the human gut and underscore the translational bridge from animal to human-focused studies.

Although clinical data has been limited, recent progress includes a Phase 1 clinical trial evaluating ShigActive™, a lytic bacteriophage cocktail targeting clinically relevant *Shigella* spp., including *S. sonnei*, *S. flexneri*, *S. dysenteriae*, and *S. boydii*. ShigActive™ consists of five obligately lytic bacteriophages, SHSML-52-1, SHFML-11, SHSML-45, SHFML-26, and SHBML-50-1, selected for their broad lytic activity and complementary host range across multiple *Shigella* spp. In this randomized, double-masked, placebo-controlled study, participants received oral ShigActive™ at a dose of approximately 1 × 10^10^ PFU per administration, three times daily for 7 days. Phages were co-administered with sodium bicarbonate to enhance gastric survival and improve intestinal delivery. Pharmacokinetic analysis demonstrated detectable phage levels in stool in 87.5% of treated subjects, with concentrations ranging from 2.0 × 10^3^ to 5.1 × 10^6^ PFU/g, confirming successful intestinal delivery and persistence. No phages were detected in blood samples, supporting localized gastrointestinal activity without systemic dissemination. These findings demonstrate that oral phage dosing at high titers and repeated administration can achieve stable intestinal exposure while maintaining an excellent safety profile and preserving gut microbiota composition [50,54].

A potential challenge for phage therapy is the emergence of phage-resistant *Shigella* variants. However, studies consistently find that when *Shigella* develops resistance to phages, it often suffers reduced fitness, reduced biofilm formation, diminished colonization capacity, or, in some cases, greater antibiotic sensitivity, as summarized in Table 1 [55].

### Oral Delivery Systems and Stability—Gut PK/PD Considerations

Oral delivery of phages is the most feasible method for obtaining high local exposure of phages with limited systemic distribution, because the initiation of a shigellosis infection in the colon is luminal. However, since phages are vulnerable to the physicochemical conditions of the upper gastrointestinal tract, there will be difficulties achieving adequate levels of phages at the site of infection. Many phages may be inactivated due to the acidic environment of the stomach when the patient is fasting, but this can change postprandially because of the transient nature of buffering of gastric acid. There will also be additional losses of phages in the duodenum and jejunum due to the presence of bile salts and other factors associated with digestion. Furthermore, spatial barriers in the intestine (mucus, crypt microhabitats, and biofilm refuges) will also limit access to target bacteria due to their location on the intestinal wall [56].

From a pharmaceutical point of view, successful oral delivery to the colon generally necessitates a protection-release strategy. The key elements to this strategy are as follows: (i) protection from gastric acid through buffering and/or an enteric, pH-dependent coating; (ii) controlled release from an intestinal environment; and (iii) enhancing the residence time with mucoadhesive microcapsules or hydrogel matrices to reduce their rapid passage through the intestine and to provide spatial refuges. By utilizing pH-triggered coatings (designed to dissolve above gastric pH) and polymer/hydrogel systems, phage recovery from simulated gastric fluid and bile salts can be maximized; in addition, these systems permit distal (throughout the small intestine and colon) phage delivery [57,58,59]. Accordingly, clinically pragmatic approaches aim to maximize the fraction of viable phage reaching the distal intestine by using buffered co-administration (e.g., sodium bicarbonate), food-timing strategies, or protective formulations such as enteric coatings and microencapsulation systems (e.g., alginate-chitosan-based matrices) that improve survival in simulated gastric fluid and enable release in intestinal conditions [56,60]. Rapid movement through the gastrointestinal tract due to diarrhea requires consideration of rapid movement through revision, dilution, or continued presence of the pathogen in order for patients to receive the necessary number of doses per day via oral administration. A useful order-of-magnitude estimate illustrates this logic: a single 10^10^ PFU dose, even with 2–4 log losses across gastric and proximal small intestinal passage, may plausibly yield 10^6^–10^8^ PFU entering the distal gut, which, after dilution in luminal fluid, may correspond to approximately 10^3^–10^6^ PFU per gram of stool, consistent with fecal recovery reported in human oral dosing studies [61]. Gut phage PK/PD differs from antibiotics because efficacy depends on both delivered dose and the capacity for in situ amplification. “Passive therapy” relies mainly on administered phage particles, whereas “active therapy” depends on phage replication that occurs only above a bacterial density threshold (proliferation threshold) [51,62]. In clinical implementation, this translational logic can be operationalized through standardized phagograms integrated into routine stool diagnostics, with defined turnaround times and decision points.

## 6. Safety and Regulatory Challenges

The advancement of bacteriophage therapy for shigellosis poses significant safety, regulatory, and efficacy challenges. Although bacteriophages are natural and abundant components of the human gut virome, their use as therapeutic agents requires careful evaluation within contemporary clinical and regulatory frameworks [63,64,65].

From a safety perspective, lytic bacteriophages are generally considered to have a favorable profile, particularly compared with broad-spectrum antibiotics. Their intrinsic specificity limits off-target effects on commensal microbiota, and their inability to infect eukaryotic cells substantially reduces the risk of direct host toxicity. Nevertheless, as biologically active viral particles, bacteriophages inevitably interact with the host immune system. Both innate and adaptive immune responses to phages have been documented, including complement activation, cytokine modulation, and the generation of neutralizing antibodies [63,64]. While immunogenicity does not necessarily translate into clinical toxicity, it may influence pharmacokinetics, dosing strategies, and the feasibility of repeated administration. Consequently, immune responses to phages represent a variable that must be systematically assessed in clinical trials and accounted for in therapeutic design [64,65].

Addressing the dynamics of phage resistance is essential for effective bacteriophage therapy. Phage-resistant bacterial variants frequently emerge as an expected outcome of phage-host coevolution, as observed across experimental systems [66,67]. Unlike AMR, phage resistance typically involves alterations in bacterial surface structures that serve as specific phage receptors. In *Shigella* spp., these receptors most commonly include components of the LPS, particularly the O-antigen polysaccharide, which represents the primary binding site for many *Shigella*-specific bacteriophages. Structural modifications, truncation, or complete loss of the O-antigen can impair phage adsorption and confer resistance. Additionally, alterations in the LPS core oligosaccharide may interfere with phage attachment. Some *Shigella* phages also recognize outer membrane proteins, including porins such as OmpA and OmpC, which may serve as secondary or alternative receptors [17,18]. Less frequently, other surface glycoconjugates may contribute to phage attachment. These receptor modifications often confer resistance but may impose fitness costs, including reduced virulence, impaired host cell invasion, or decreased ecological competitiveness. However, the dynamics of phage resistance within the complex gut ecosystem remain poorly understood, especially under therapeutic conditions that often differ from those in laboratory models. The long-term clinical effects of phage resistance, including its influence on treatment durability, require further investigation through longitudinal studies and real-world clinical studies [66,67].

Additional concerns relate to the potential for horizontal gene transfer (HGT) in the context of phage therapy. Certain bacteriophages, particularly temperate phages, can mediate transduction and integrate into bacterial genomes, thereby contributing to lysogenic conversion or the dissemination of virulence and AMR genes [68]. Contemporary therapeutic strategies, however, rely almost exclusively on strictly lytic phages, and advances in whole-genome sequencing now enable comprehensive screening for genes associated with lysogeny, toxin production, or resistance determinants prior to clinical use [68,69]. Although the risk of HGT cannot be entirely eliminated, rigorous genomic characterization and careful phage selection substantially mitigate this concern and are now considered essential prerequisites for regulatory approval [68,69]. Immunocompromised individuals represent a high-risk population for severe and prolonged shigellosis, and immune status may significantly influence bacteriophage pharmacokinetics, efficacy, and safety. Host immunity plays a key role in modulating phage clearance, primarily through reticuloendothelial system uptake and the production of neutralizing antibodies [70,71]. In immunocompromised patients, reduced phage neutralization and slower systemic clearance may prolong phage persistence, potentially enhancing therapeutic exposure and efficacy [70]. However, impaired immune function may also reduce synergistic interactions between bacteriophages and host innate immunity, which normally contribute to bacterial clearance [71,72]. Additionally, alterations in mucosal immunity, particularly in individuals with HIV infection, hematological malignancies, or immunosuppressive therapy, may affect phage distribution and activity at intestinal sites of infection [72]. From a safety perspective, bacteriophages are generally considered safe due to their high specificity and lack of direct toxicity to human cells; however, altered immune responses and reduced phage clearance in immunocompromised hosts warrant careful evaluation [70,71,72]. Overall, while phage therapy may offer particular advantages in immunocompromised individuals due to its targeted antibacterial activity and independence from conventional antibiotic mechanisms, further clinical studies are needed to characterize pharmacokinetics, optimal dosing strategies, and safety profiles in this vulnerable population.

Substantial regulatory challenges hinder the broad adoption of phage therapy in clinical practice. Existing regulatory frameworks were largely developed for chemically defined pharmaceuticals and are often poorly suited to biologically adaptive agents such as bacteriophages. Unresolved issues include product standardization, batch-to-batch consistency, stability testing, and quality control, particularly in the context of phage cocktails or personalized formulations [73]. Moreover, conventional clinical trial designs may not fully capture key features of phage therapy, such as pathogen specificity, self-amplification at the site of infection, and dynamic interactions with bacterial populations [74]. As a result, there is growing recognition of the need for tailored regulatory pathways and harmonized international guidelines to ensure robust clinical evaluation and consistent assessment of safety and efficacy [73,74,75]. A *Shigella*-specific regulatory challenge relates to the distinction between therapeutic use in acute dysentery and decolonization strategies in asymptomatic carriers or convalescent individuals. In acute shigellosis, rapid pathogen clearance is essential in order to reduce disease severity, prevent systemic complications, and limit transmission, particularly in vulnerable populations such as children, elderly individuals, and immunocompromised patients [76]. In this context, regulatory evaluation must prioritize clinical efficacy endpoints, including time to symptom resolution, reduction in bacterial shedding, and prevention of complications. In contrast, decolonization strategies aim to eliminate persistent intestinal carriage while preserving microbiota stability and minimizing unintended ecological disruption. For these indications, safety, microbiota preservation, and long-term ecological effects become primary regulatory considerations, particularly given the ecological role of commensal microbiota in colonization resistance and pathogen exclusion [77]. This distinction has important implications for clinical trial design, endpoint selection, risk-benefit assessment, and regulatory approval pathways, especially in light of the dual clinical and public health objectives of treating symptomatic infection and interrupting transmission chains [78]. Therapeutic frameworks must therefore account for the fundamentally different clinical objectives of rapid antimicrobial intervention vs. microbiological decolonization, particularly for enteric pathogens such as *Shigella*, where both acute disease management and transmission control represent critical public health priorities. In the context of the European Medicines Agency and the United States Food and Drug Administration, concrete translational pathways are beginning to emerge, including adaptive regulatory routes such as compassionate use programs, expanded access programs, and magistral or personalized bacteriophage preparations produced under good manufacturing practice conditions. Both agencies increasingly emphasize whole-genome characterization, validated manufacturing processes, and pathogen-specific clinical endpoints, while allowing flexible frameworks for bacteriophage cocktails and iterative updates of bacteriophage composition [79,80]. These evolving regulatory models are expected to accelerate the controlled clinical integration of bacteriophage therapy for antibiotic-resistant enteric infections, including shigellosis.

Integrating immunological and regulatory factors is crucial for translating bacteriophage therapy into clinical use. Continued genomic safety screening, immune assessment, and clinical trials, supported by updated regulatory frameworks, will help define the role of bacteriophage therapy in modern antimicrobial approaches.

## 7. Future Perspectives: Toward Personalized Phage Therapy in Gastroenterology Field

In the field of gastroenterology, personalized phage therapy is evolving from an appealing concept to a systemic problem: can a strain-targeted antimicrobial be administered with the speed and repeatability needed in actual clinical workflows? Many pathobiont-driven conditions and gastrointestinal infections are strain-specific, niche-specific, and change over time. Consequently, the field is coming to a consensus on a platform model that includes quick identification of the offending strain, quick matching to a carefully selected phage set, and logical combination strategies that predict bacterial escape [81].

### 7.1. Phage Cocktails and Personalized Phage Selection

The first challenge, matching, is caused by the same host specificity that makes phages microbiota-sparing. Finding phages that are active against the patient’s isolate (rather than just the species) is essential for effective use, and this still calls for screening and characterization to verify activity [82]. Efficiency-of-plating (EOP) assays and spot tests are commonly used in practice to estimate virulence and host range, but EOP is more labor-intensive and more discriminating than spot tests, which can overestimate both [83]. These limitations force the field to move away from ad hoc decisions and toward standardized selection processes. The most sensible solution to single-step escape and within-species diversity is still cocktails. Cocktail building, however, is not just additive: assembling several phages takes time, and careful selection is required to prevent combinations that lower overall performance [84]. Cocktail logic is becoming more and more concerned with covering subpopulations and microhabitats rather than just expanding “spectrum” in gastroenterology, where bacterial populations can be spatially heterogeneous (mucus-associated, biofilm-associated, lumen-dominant).

### 7.2. Integration with Metagenomics and Precision Medicine

Precision diagnostics will increasingly be used in conjunction with precision phage therapy. When combined with strain-resolved methods, metagenomic profiling can facilitate easier target identification, identify mixed infections or strain replacement, and assist in distinguishing between reinfection and persistence in recurrence. Metagenomics does not replace phenotypic susceptibility testing in a practical pipeline; rather, it enhances it by indicating which isolates and niches are important and when re-matching is necessary [85]. Additionally, this diagnostic layer is in line with the more general precision direction in gastroenterology, which bases therapy selection on ecological context, recurrence risk, and microbial signatures rather than just syndrome labels.

### 7.3. Artificial Intelligence and Phage Discovery Platforms

Delivery time, rather than concept, is frequently the limiting factor for personalization.

The primary way in which treatment for *Shigella* may benefit from artificial intelligence (AI) derives from refining receptor-level matching. *Shigella* viruses frequently exhibit restricted host ranges due to variations in O-antigens and other external markers. Proteins, including those from the Sfk20 virus, can be analyzed through proteomics and structure modeling using AlphaFold2 and Phyre2 to provide higher-quality predictions of protein structure compared to ESMFold, thereby supporting the identification and ranking of likely candidate host-binding proteins for subsequent investigation [86]. In a practical workflow, predicted receptor binding protein (RBP) modules can be clustered and linked to bacterial surface genotypes (e.g., O-antigen locus variation) to generate hypotheses about adsorption targets and to rank candidate phages for a given clinical isolate. This does not replace wet-lab confirmation, but it can narrow the screening space, accelerate cocktail design under receptor diversity constraints, and support rational updates when strain replacement or adsorption-based resistance emerges [87,88].

Protein language models, including ProtT5, and structure-aware models, such as SaProt, allow for generating embeddings to integrate sequence, and in some cases structural, embedding information. These embedding-based approaches have demonstrated improved performance on predicting phage-host interactions, particularly on RBPs, which are the key determinants of host specificity. Programmatically generated embedding approaches outperform previous feature-engineered approaches and sequence similarity-based approaches, especially given the common occurrence (like in phage genomic sequences) of low sequence similarity between training and testing proteins to predict which proteins will bind phages. Overall, structure-aware embedding approaches yield higher predictive performance than comparable sequence similarity examples when the sequence similarity between training and testing is less than 40%, again indicating the advantage of using structural information to accurately predict hosts that will ultimately support the phage growth [87,88].

Predicting host range, ranking candidate phages for a particular bacterial genome, and optimizing cocktails under limitations like receptor diversity and manufacturability are examples of AI-enabled discovery and matching platforms that are being developed to narrow the search space and speed up decisions [89,90]. Crucially, triage and acceleration, rather than replacing wet-lab confirmation, are the most practical applications of AI. Compressing the match-to-treatment timeline is likely to be the key benefit of these platforms in a gastrointestinal setting where recurrence windows can be brief and clinical deterioration can occur quickly.

### 7.4. Roadmap for Clinical Translation

A workable roadmap has four operational steps. First, standardize the clinical interface: implement routine isolate handling, rapid susceptibility workflows, and comparable reporting across centers, with standardized “phagograms” and automated testing systems as the backbone [91].

Standardized phagograms should fit within the existing enteric diagnostic workflow instead of operating independently. A typical process would be to use: (i) same day multiplex stool polymerase chain reaction (PCR, or rapid molecular testing) for confirmation of an invasive enteric pathogen signature so that the immediate decision regarding infection control and empiric therapy can be made; (ii) perform concurrent stool culture with isolate archiving to allow future confirmation of the phage match; and (iii) utilize a phased approach to the phagogram after an isolate has become available [92]. As such, turnaround time for PCR confirmation is gauged in hours and can be as much as 24–48 h (culture and isolate recovery) plus an additional 12–24 h for standardized susceptibility testing (rapid screen by spot test followed by EOP confirmation of candidate phages or cocktails) [93,94,95]. This creates two clinical decision points: an early decision (day 0) focused on diagnosis and risk stratification, and a second decision (day 2–3) when isolate-linked phage susceptibility data allow targeted phage selection or cocktail tailoring [96]. Integrating PCR/culture outputs with susceptibility testing is the best way to handle co-infections: phage therapy should focus on the main clinical pathogen (or a confirmed *Shigella* isolate), while the co-infecting pathogens will be treated according to standard-of-care with broad or modular phage cocktails if multiple relevant strains are identified [97]. Enteric ecosystems have a dynamic nature and therefore require clear retriggering protocols for being re-tested, such as lack of clinical response, recurrence, or microbiological evidence of strain replacement (new serotype/strain from follow-up cultures or strain resolved from sequencing). Phagograms must also be re-evaluated on the new isolate, and the composition of cocktails will be updated through an iterative and precise workflow for each occasion [98].

Second, scale personalization through curated libraries and modular cocktails: select phages using reproducible criteria, then assemble rational combinations that broaden coverage and reduce the probability of escape, acknowledging that developing effective cocktails requires time and careful design [91]. Third, embed re-testing into follow-up: because bacterial populations can shift, personalization should be treated as an iterative process, with re-matching triggered by recurrence, strain replacement, or reduced response. Fourth, build production-ready pipelines that can deliver clinically usable preparations within meaningful timeframes; this is where mechanization and standardization become non-negotiable, given that current phage susceptibility testing remains labor-intensive and time-consuming [99].

Low- and middle-income countries bear a high level of burden from shigellosis, making manufacturing costs and distribution logistics factors involved in clinical feasibility rather than optional optimizations. The public health impact of precision matching and tailored cocktails will be limited if the final products cannot be manufactured in sufficient quantities at an acceptable cost per course of treatment [100]. Thus, the implementation of the roadmap must focus on developing manufacturing strategies that enable large-scale (high throughput) and consistent (standardized) production of oral products using common elements (such as robust host strains) while considering the needs of stability and cold chain for oral products produced this way. A realistic long-term objective is to create “precision capable” platforms (with respect to how they are assembled) including libraries and modular cocktails, which still depend on reliable and cost-controlled manufacturing processes that can be utilized in resource-poor environments.

Across these steps, the central theme is scalability without losing precision. The field’s success in the gastroenterology field will depend on whether personalization can be made routine: fast matching, rational cocktails, standardized testing, and iterative updating. The detailed safety, resistance biology, and regulatory frameworks that shape implementation are addressed elsewhere; the future perspective here is that these constraints ultimately define the design requirements for a clinically usable personalization platform. Key translational barriers and required advancements for clinical implementation of bacteriophage therapy are summarized in Table 2.

## 8. Conclusions

Shigellosis sits at the intersection of escalating AMR and microbiota-dependent disease vulnerability, exposing the limits of antibiotic-centric management. In this context, bacteriophage therapy should not be viewed as a rescue option, but as a rational re-alignment of treatment with pathogen ecology. Cumulative evidence indicates that *Shigella* is biologically well suited to phage targeting, with surface dependencies, fitness trade-offs, and biofilm susceptibility that favor durable control rather than simple bacterial suppression. Crucially, phages achieve pathogen reduction without compounding gut dysbiosis, preserving colonization resistance that antibiotics routinely erode. The central challenge is no longer proof of concept, but implementation: rapid diagnostics, standardized phage selection, and regulatory frameworks adapted to adaptive biologics. Addressed decisively, phage therapy could redefine shigellosis management as a precision, microbiota-conserving intervention rather than a blunt antimicrobial compromise.

## Figures and Tables

**Table 1:** Preclinical and clinical evidence regarding bacteriophage therapy against *Shigella* spp.

Study	Model	Phages Evaluated	Main Findings	Key Translational Implications
Mai et al. [50]	Murine model of *Shigella* intestinal infection	Oral phage cocktail	Significant reduction in intestinal colonization and fecal shedding; efficacy comparable to ampicillin; no detectable toxicity; preservation of gut microbiota diversity.	Proof of concept for oral phage therapy; microbiota-sparing antimicrobial activity compared with antibiotics.
Mondal et al. [51]	In vitro assays and human cell lines	Lytic phage Sspk23	Strong lytic activity against *S. sonnei*; effective disruption of established biofilms; no cytotoxicity in intestinal epithelial and macrophage-like cell lines.	Demonstration of biofilm-targeting capacity and favorable in vitro safety profile.
Mondal et al. [52]	Murine model (*S. sonnei*-infected BALB/c mice)	Lytic phage SSG23	Broad host range covering all four *Shigella* spp.; high stability across pH and temperature ranges; absence of toxin or resistance genes; significant reduction in bacterial burden and shedding; maintained efficacy despite antibody development.	Strong in vivo efficacy and genomic safety; supports feasibility of oral administration.
Llanos-Chea et al. [53]	Human intestinal organoid-derived epithelial monolayers	*S. flexneri*-specific phage	Efficient killing of *Shigella*; prevention of epithelial adherence and invasion; strict pathogen specificity; no effect on commensal *Escherichia coli.*	Mechanistic insight into interference with early pathogenic steps; high translational relevance.
Chen et al. [54]	Phase 1 randomized, double-masked, placebo-controlled clinical trial	Lytic phage cocktail targeting all *Shigella* spp.	Good tolerability and safety after oral administration; no serious adverse events; no significant changes in inflammatory biomarkers or gut microbiota composition.	Evidence of safety and feasibility in humans.
Zhao et al. [55]	In vitro and in vivo experimental models	Various *Shigella*-specific phages	Decreased biofilm formation, impaired colonization capacity, and increased antibiotic susceptibility in some strains.	Phage resistance may impose biological costs and attenuate pathogenicity rather than confer a selective advantage.

**Table 2:** Key translational barriers to clinical implementation of bacteriophage therapy and current versus required solutions.

Translational Barrier	What Exists Now	What Is Needed
Immunogenicity and host immune clearance	Clinical trials demonstrate favorable safety profiles, but phages may be neutralized by circulating antibodies or cleared by the reticuloendothelial system, potentially reducing persistence and efficacy. Limited pharmacokinetic data are available for enteric applications.	Improved characterization of phage pharmacokinetics and immune interactions, optimized dosing regimens, and formulation strategies (e.g., encapsulation or buffered delivery) to enhance intestinal persistence and therapeutic effectiveness.
HGT risk and genomic safety	Whole-genome sequencing is routinely used to exclude lysogenic phages and those carrying virulence or AMR genes. Obligate lytic phages are preferentially selected for therapeutic use.	Standardized genomic screening pipelines, internationally harmonized safety criteria, and real-time monitoring systems to minimize HGT risk and ensure long-term genomic stability of therapeutic phage
Manufacturing, scalability, and quality control	Small-scale production of clinical-grade bacteriophage preparations is feasible, but manufacturing remains labor-intensive and difficult to scale. Batch-to-batch variability and the absence of universally standardized production and purification workflows limit broader clinical implementation.	Scalable, automated production platforms with standardized purification, sterility assurance, and quality control procedures to ensure consistency, safety, reproducibility, and timely availability of therapeutic phage preparations.
Regulatory pathway and clinical framework	Regulatory pathways are emerging but remain heterogeneous across jurisdictions. Phage therapy is often regulated under compassionate use, magistral preparation, or experimental frameworks.	Clear, harmonized regulatory frameworks defining classification, approval pathways, quality standards, and clinical trial requirements to support routine clinical adoption.
Diagnostic infrastructure and susceptibility testing	Phage susceptibility testing (“phagograms”) is technically feasible but not standardized and remains limited to specialized laboratories. Turnaround times may delay therapeutic decision-making.	Rapid, automated, and standardized phage susceptibility testing platforms integrated into clinical microbiology workflows to enable timely personalization and routine clinical use.

Abbreviations: HGT, horizontal gene transfer; AMR, antimicrobial resistance.

## Data Availability

No new data were created or analyzed in this study. Data sharing is not applicable to this article.

## Abbreviations

The following abbreviations are used in this manuscript:

AMR	Antimicrobial resistance
AI	Artificial intelligence
EOP	Efficiency of plating
HGT	Horizontal gene transfer
LPS	Lipopolysaccharide
MDR	Multidrug-resistant
PCR	Polymerase chain reaction
PFU	Plaque-forming unit
RBP	Receptor binding protein
SCFAs	Short-chain fatty acids
